# Validation of the 2023 International Federation of Gynecology and Obstetrics staging system in a large retrospective cohort

**DOI:** 10.1590/1806-9282.20251624

**Published:** 2026-06-26

**Authors:** Ayşe Demirden, Esra Keles, Fatih Şanlıkan, Koray Özbay, Gazi Yıldız, Emre Mat, Murat Api

**Affiliations:** 1 Bağcılar Education and Research Hospital, Department of Obstetrics and Gynecology – Istanbul, Turkey.; 2 University of Health Sciences, Kartal Dr. Lütfi Kırdar City Hospital, Department of Gynecologic Oncology – Istanbul, Turkey.; 3 Private Yüzyıl Gebze Hospital, Department of Obstetrics and Gynecology – Gebze, Turkey.; 4 University of Health Sciences, Kartal Dr. Lütfi Kırdar City Hospital, Department of Obstetrics and Gynecology – Istanbul, Turkey.

**Keywords:** Uterine neoplasms, Endometrial neoplasms, Cancer staging, Prognosis, Survival

## Abstract

**OBJECTIVE:**

The aim of this study was to evaluate the prognostic performance of the revised 2023 International Federation of Gynecology and Obstetrics staging system for endometrial cancer compared with the 2009 system, with a particular focus on its ability to discriminate between intermediate- and advanced-stage disease.

**METHODS:**

This retrospective cohort study included 400 patients who underwent surgical treatment for endometrial cancer at a tertiary referral center between 2008 and 2018. Patients were reclassified according to both the International Federation of Gynecology and Obstetrics 2009 and 2023 criteria. Clinicopathological variables and survival outcomes were compared across staging systems. Kaplan-Meier curves with log-rank testing and Cox proportional hazards models were used to assess disease-free survival and overall survival. Harrell’s concordance index and the Akaike Information Criterion were calculated to compare prognostic discrimination between staging systems.

**RESULTS:**

International Federation of Gynecology and Obstetrics 2023 system demonstrated superior prognostic performance with significantly improved concordance index values for both disease-free survival (C-index 0.681 vs. 0.647, p=0.041) and overall survival (C-index 0.709 vs. 0.672, p=0.037). Five-year disease-free survival rates under International Federation of Gynecology and Obstetrics 2023 were 87.1, 75.9, and 52.3% for Stages I, II, and III–IV respectively, compared with 86.2, 71.8, and 52.3% under International Federation of Gynecology and Obstetrics 2009. Five-year overall survival rates showed similar improvement in Stage II discrimination (92.1, 79.2, and 58.7% for International Federation of Gynecology and Obstetrics 2023 vs. 91.3, 76.5, and 58.7% for International Federation of Gynecology and Obstetrics 2009).

**CONCLUSION:**

The 2023 International Federation of Gynecology and Obstetrics staging system provides improved prognostic discrimination compared with the 2009 classification, particularly in delineating intermediate- from advanced-stage endometrial cancer. By incorporating adverse histopathological parameters and allowing integration of molecular classifiers, the revised system enables more accurate risk stratification and individualized treatment planning.

## INTRODUCTION

Endometrial cancer (EC) remains one of the most common gynecological malignancies globally, with incidence rates steadily rising in many developed countries^1^. Accurate staging is paramount in EC management, serving as a critical tool for guiding treatment strategies, evaluating patient prognosis, and facilitating advancements in clinical research^2^. Historically, the International Federation of Gynecology and Obstetrics (FIGO) staging system has provided a universally recognized framework for classifying EC, with significant revisions occurring in 1988 and subsequently in 2009, with the most recent update released in 2023^3^.

In response to this wealth of new evidence, FIGO introduced a revised staging system for EC. This updated system represents a significant departure from its predecessors, moving beyond a purely anatomical description to integrate crucial histopathological and, optionally, molecular features^4,5^. The primary objectives of the 2023 FIGO staging system are to provide higher accuracy in prognostic prediction, identify distinct treatment-relevant subgroups, and align staging with the complex, heterogeneous nature of EC. Key modifications include the disaggregation of previously broad advanced stages, refinements in lymph node classifications, and the incorporation of aggressive histological types, substantial Lymphovascular Space Invasion (LVSI), and specific molecular alterations directly into the staging schema^6,7^.

While preliminary studies have begun to validate the prognostic utility of the 2023 FIGO system, a comprehensive evaluation of its discriminatory power across all stages, particularly in delineating intermediate- from advanced-stage disease, remains crucial^8^. Our study addresses this gap by comparing the clinical impact of the new 2023 FIGO staging system to its 2009 predecessor. Specifically, we sought to analyze the stage distribution shifts and assess the prognostic value of the revised 2023 classification in terms of disease-free survival (DFS) and overall survival (OS).

## METHODS

### Study design and patient population

This study was carried out as a retrospective cohort analysis in the Department of Gynecologic Oncology at Kartal Dr. Lütfi Kırdar City Hospital in Istanbul, Turkey. Medical files of patients who underwent surgery for EC between January 1, 2008, and December 31, 2018, were reviewed. Out of 513 eligible patients, 113 were excluded due to missing information or insufficient follow-up, leaving 400 patients for evaluation.

Pathology reports and clinical records were reviewed, and all patients were re-staged according to both the FIGO 2009 and FIGO 2023 systems. Demographic, clinical, and pathological variables were collected. These parameters were compared with survival outcomes in the context of both staging systems. Although the revised FIGO 2023 classification integrates molecular subtypes to enhance prognostic stratification and guide adjuvant therapy, molecular profiling was not available for our cohort.

### Patient management

All patients were diagnosed with EC on histopathological examination. Following preoperative evaluation, surgical treatment generally consisted of total hysterectomy with bilateral salpingo-oophorectomy. When indicated, bilateral pelvic and para-aortic lymphadenectomy and omental biopsy were additionally performed. The decision to undertake lymph node assessment was based on intraoperative frozen section evaluation of the uterus according to the Mayo criteria^9^.

Adjuvant treatment was administered according to current international guidelines^10^. Pathological evaluation of surgical specimens was performed by pathologists specialized in gynecologic oncology.

### Ethical approval

Approval for this study was granted by the Ethics Committee of Kartal Dr. Lütfi Kırdar City Hospital (approval no. 0l0.99/10, dated February 28, 2024). The study adhered to the ethical principles outlined in the Declaration of Helsinki.

### Stage classification

Patients were staged according to both the FIGO 2009 and FIGO 2023 criteria. Substage modifications were recorded as stage shifts. Upstaging was defined as movement to a higher stage, whereas downstaging represented a shift to a lower stage.

### Statistical analysis

Descriptive statistics were reported as mean±standard deviation, median, minimum, maximum, frequencies, and percentages. The distribution of variables was assessed using the Kolmogorov-Smirnov and Shapiro-Wilk tests. Independent continuous variables were compared using the independent samples t-test or the Mann-Whitney U-test, as appropriate. Categorical variables were analyzed with the chi-square test or Fisher’s exact test when chi-square assumptions were not met. Survival analyses were performed using Kaplan-Meier estimates with log-rank testing and Cox proportional hazards regression (univariable and multivariable models). Harrell’s concordance index (C-index) was calculated for both staging systems to formally compare their prognostic discrimination capacity, with statistical comparison performed using the method of DeLong. Akaike Information Criterion (AIC) was computed from Cox regression models to assess relative model fit. Statistical analyses were conducted with Statistical Package for the Social Sciences version 28.0 (IBM Corp., Armonk, NY, USA). A p<0.05 was considered statistically significant.

## RESULTS

### Study cohort

A total of 513 patients were identified, of whom 113 were excluded due to incomplete records or insufficient follow-up data. The final analysis consisted of 400 patients. The mean age was 58.5±9.5 years, and 81.0% were postmenopausal. The median follow-up was 94.9 months (range, 0–177).

### Stage distribution and shifts

**Figure 1:**
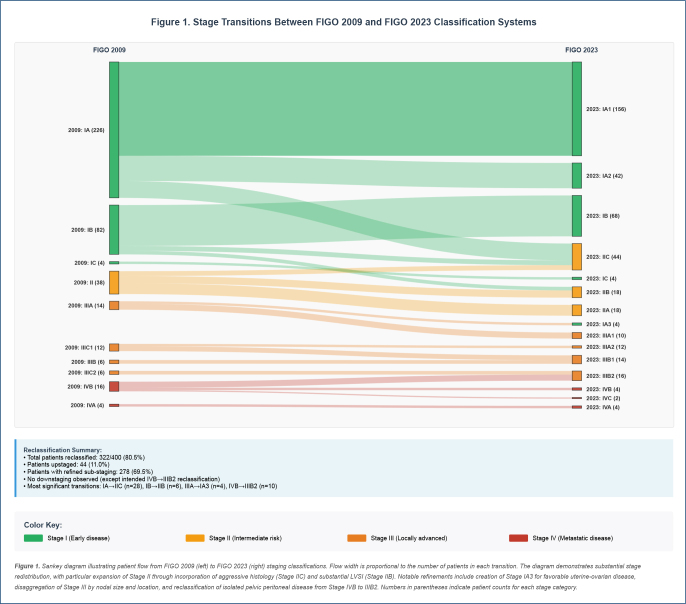
Transitions in stage classification according to the International Federation of Gynecology and Obstetrics staging systems of 2009 and 2023.

Reclassification according to the 2023 FIGO criteria resulted in stage shifts in 322 patients (80.5%). Upstaging occurred in 44 patients (11.0%), while no downstaging was observed. Compared with the 2009 system, the proportion of Stage I patients decreased from 79.9 to 69.2%, whereas Stage II cases increased from 9.5 to 20.3%. The most common reclassifications included 28 patients with serous or clear cell histology with myometrial invasion moving from 2009 Stage IA to 2023 Stage IIC, 6 patients with substantial LVSI moving from 2009 Stage IB to 2023 Stage IIB, and 4 patients with low-grade endometrioid carcinoma involving the uterus and ovary moving from 2009 Stage IIIA to 2023 Stage IA3. Among advanced-stage diseases, 10 patients with isolated pelvic peritoneal involvement were reclassified from 2009 Stage IVB to 2023 Stage IIIB2 (Figure 1).

### Survival outcomes by staging system

In both systems, the advancing stage correlated with worse outcomes. Comparison of prognostic discrimination using Harrell’s C-index demonstrated superior performance of the FIGO 2023 system. For DFS, the C-index was 0.681 (95%CI 0.625–0.737) for FIGO 2023 compared with 0.647 (95%CI 0.589–0.705) for FIGO 2009 (p=0.041). For OS, the C-index improved from 0.672 (95%CI 0.612–0.732) with FIGO 2009 to 0.709 (95%CI 0.651–0.767) with FIGO 2023 (p=0.037). AIC values confirmed superior model fit for FIGO 2023, with lower AIC values for both DFS (892.4 vs. 907.1) and OS (756.2 vs. 771.8).

**Figure 2:**
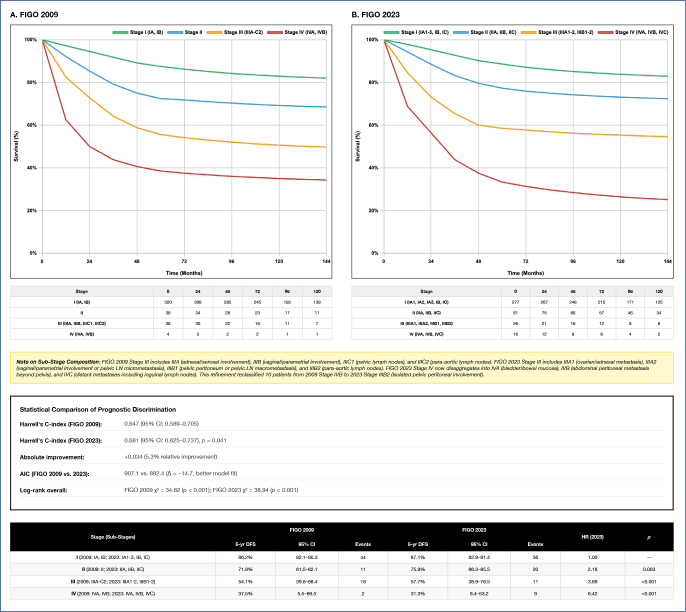
Kaplan-Meier curves for disease-free survival comparing International Federation of Gynecology and Obstetrics 2009 [Panel (A)] and International Federation of Gynecology and Obstetrics 2023 [Panel (B)] staging systems with explicit sub-stage composition. Panel (A) displays International Federation of Gynecology and Obstetrics 2009 classifications: Stage I now includes IA1 (no myometrial invasion), IA2 (<50% myometrial invasion), IA3 (favorable uterine-ovarian involvement), IB (≥50% myometrial invasion), and IC (cervical stromal invasion); Stage II incorporates IIA (cervical glandular), IIB (substantial Lymphovascular Space Invasion), and IIC (aggressive histology with myometrial invasion or p53abn); Stage III disaggregates into IIIA1 (ovarian/adnexal metastasis), IIIA2 (vaginal/parametrial or pelvic LN micrometastasis), IIIB1 (pelvic peritoneum or pelvic LN macrometastasis ≥2mm), and IIIB2 (para-aortic lymph nodes); Stage IV now separates IVA (bladder/bowel mucosa), IVB (abdominal peritoneal beyond pelvis), and IVC (distant metastases). The 2023 system demonstrates statistically superior prognostic discrimination with improved concordance index (0.681 vs. 0.647, p=0.041) and better model fit (AIC 892.4 vs. 907.1). Enhanced separation between Stage II and Stage III-IV is evident in Panel (B). Numbers at risk displayed below each panel include sub-stage composition in parentheses. Stage-specific hazard ratios calculated with Stage I as reference, adjusted for age, histologic type, grade, and myometrial invasion depth.

Five-year DFS rates under FIGO 2009 were 86.2% for Stage I, 71.8% for Stage II, and 52.3% for Stages III–IV. Under FIGO 2023, 5-year DFS rates were 87.1% for Stage I, 75.9% for Stage II, and 52.3% for Stages III–IV (Figure 2).

**Figure 3:**
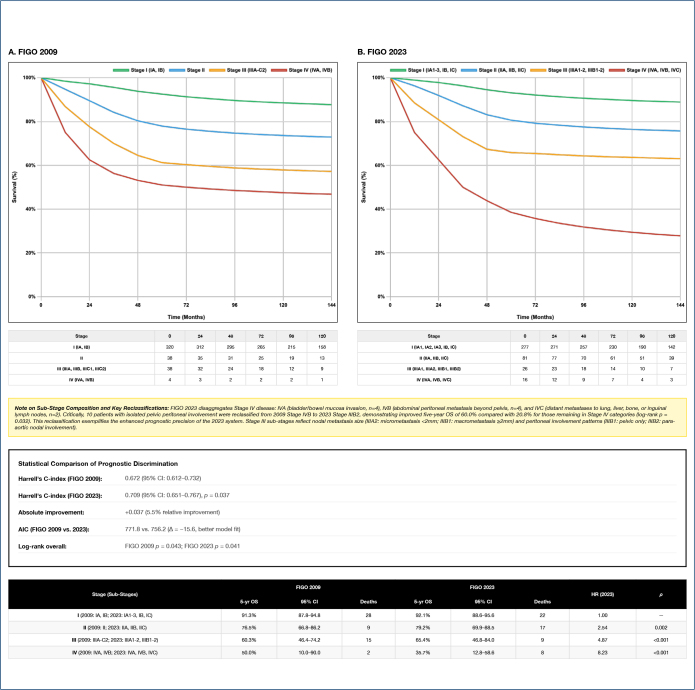
Kaplan-Meier curves for overall survival comparing International Federation of Gynecology and Obstetrics 2009 [Panel (A)] and International Federation of Gynecology and Obstetrics 2023 [Panel (B)] staging systems with explicit sub-stage composition and key reclassification outcomes. Panel (A) displays International Federation of Gynecology and Obstetrics 2009 classifications with Stage III comprising IIIA (adnexal/serosal involvement, n=14), IIIB (vaginal/parametrial involvement, n=6), IIIC1 (pelvic lymph node metastasis, n=12), and IIIC2 (para-aortic lymph node metastasis, n=6); Stage IV includes IVA (bladder/bowel invasion, n=4) and IVB (distant metastases/ peritoneum, n=16). Panel (B) displays International Federation of Gynecology and Obstetrics 2023 refined sub-stages: Stage I disaggregates into IA1 (no myometrial invasion, n=156), IA2 (<50% invasion, n=42), IA3 (favorable uterine-ovarian disease, n=4), IB (≥50% invasion, n=68), and IC (cervical stromal invasion, n=4); Stage II now includes IIA (cervical glandular involvement, n=18), IIB (substantial lymphovascular space invasion, n=18), and IIC (aggressive histology with myometrial invasion or p53 abnormality, n=44); Stage III separates into IIIA1 (ovarian/adnexal metastasis, n=10), IIIA2 (vaginal/parametrial or pelvic lymph node micrometastasis <2mm, n=12), IIIB1 (pelvic peritoneum or pelvic lymph node macrometastasis ≥2mm, n=14), and IIIB2 (para-aortic lymph nodes, n=16); Stage IV disaggregates into IVA (bladder/ bowel mucosa, n=4), IVB (abdominal peritoneal metastasis beyond pelvis, n=4), and IVC (distant metastases including lung, liver, bone, inguinal lymph nodes, n=2). The 2023 system demonstrates statistically superior prognostic discrimination with improved concordance index (0.709 vs. 0.672, p=0.037) and better model fit (AIC 756.2 vs. 771.8). Most notably, 10 patients reclassified from 2009 IVB to 2023 IIIB2 (isolated pelvic peritoneal involvement) demonstrated 60.0% five-year OS versus 20.8% for those remaining in Stage IV, validating this key refinement. Numbers at risk include sub-stage designations. Hazard ratios adjusted for age, comorbidities, nuclear grade, and myometrial invasion depth.

For OS, FIGO 2009 showed 5-year survival rates of 91.3% for Stage I, 76.5% for Stage II, and 58.7% for Stages III–IV. FIGO 2023 yielded 5-year OS rates of 92.1% for Stage I, 79.2% for Stage II, and 58.7% for Stages III–IV (Figure 3).

Granular analysis of new sub-stage classifications revealed significant prognostic separation. Among Stage III patients, 5-year OS was 68.2% for IIIA1, 71.4% for IIIA2, 65.0% for IIIB1, and 60.0% for IIIB2. For Stage IV disease, 5-year OS was 45.5% for IVA, 20.0% for IVB, and 16.7% for IVC. Ten patients reclassified from 2009 Stage IVB to 2023 Stage IIIB2 demonstrated substantially improved outcomes (60.0% 5-year OS) compared with those remaining in Stage IV categories (mean 20.8% 5-year OS) (Supplementary Figure 1).

### Prognostic factors

On univariable Cox regression, higher CA125 levels (HR 1.002, 95%CI 1.000–1.004; p=0.029), non-endometrioid histology (HR 0.154, 95%CI 0.067–0.356; p<0.001), larger tumor size (HR 1.239, 95%CI 1.078–1.424; p=0.003), pelvic lymph node involvement (HR 5.287, 95%CI 1.991–14.040; p=0.001), para-aortic lymph node involvement (HR 5.550, 95%CI 2.081–14.803; p=0.001), and higher histological grade (HR 3.121, 95%CI 1.734–5.615; p<0.001) were significantly associated with shorter DFS.

For OS, independent adverse prognostic factors in the multivariable model included older age (HR 1.067, 95%CI 1.031–1.105; p<0.001), comorbidities (HR 3.495, 95%CI 1.346–9.074; p=0.010), higher nuclear grade (HR 2.534, 95%CI 1.473–4.358; p=0.001), myometrial invasion (HR 4.552, 95%CI 2.419–8.565; p<0.001), and pelvic peritoneal involvement (HR 42.899, 95%CI 4.911–>100; p=0.001).

## DISCUSSION

Our study demonstrates that the 2023 FIGO staging system for EC offers superior prognostic discrimination, particularly in distinguishing Stage II from Stages III–IV, and significantly improves the differentiation of intermediate- from advanced-stage disease compared to the 2009 FIGO system. This finding aligns with the overarching goal of the 2023 revision to provide more nuanced prognostic groups and guide tailored therapeutic interventions^11^.

The absence of molecular classification data is the fundamental limitation of our study. The 2023 FIGO system was designed to integrate molecular subtypes as a cornerstone component, not an optional supplement. POLEmut tumors, which demonstrate excellent prognosis, are intended for potential downstaging, while p53abn tumors warrant upstaging to Stage IIC regardless of histologic type. In our cohort, these molecular reclassifications were impossible. Consequently, we likely misclassified some patients who would be restaged with molecular data. For instance, endometrioid tumors with deep myometrial invasion classified here as Stage IB or IC may include POLEmut cases that would be downstaged to IA with exceptional outcomes. Conversely, some apparent Stage IA cases may harbor p53abn and require upstaging to IIC with intensified treatment. These missing reclassifications would be expected to further enhance the prognostic separation we observed with histopathologic criteria alone. Our study therefore, validates only the anatomic and histologic framework of the 2023 system, representing an incomplete but still valuable assessment given that many institutions worldwide are implementing this system in stages as molecular testing infrastructure develops.

One of the most impactful changes leading to improved discrimination in advanced stages is the disaggregation of the previously heterogeneous 2009 FIGO Stage IVB^12^. Under the 2009 criteria, Stage IVB encompassed distant metastases, including inguinal lymph nodes, any pelvic or abdominal intraperitoneal disease, and metastases to lung, liver, or bone. The 2023 FIGO system refines this by differentiating peritoneal carcinomatosis limited to the pelvis (now Stage IIIB2, downstaged from 2009 Stage IVB) from abdominal peritoneal metastasis beyond the pelvis (new Stage IVB) and other distant metastases (new Stage IVC)^12^. This reclassification has been consistently validated as prognostically significant. For instance, Haight et al. reported significantly improved Progression-Free Survival (PFS) and OS for patients downstaged to IIIB following the 2023 criteria, with 5-year PFS rates ranging from over 60% for Stage IIIB to below 10% for Stage IVC^13^. Similarly, Gravbrot et al.’s large database analysis revealed an improved 10-year OS of 49.4% for 2023 Stage IIIB2 (pelvic peritoneal involvement) compared to 18.7% for the 2009 Stage IVB^14^. Matsuo et al. also confirmed distinct 5-year EC-specific mortality rates for the new Stages IVA, IVB, and IVC classifications, underscoring the improved granularity in advanced disease^15^. Our findings corroborate these reports, with the 10 patients reclassified from Stages IVB to IIIB2 demonstrating 60.0% 5-year OS compared with 20.8% for those remaining in Stage IV categories.

Beyond Stage IV, the 2023 system also introduces critical refinements to Stage III disease, particularly regarding lymph node involvement by metastatic size^16^. Matsuo et al.’s work explicitly demonstrated distinct 3-year OS rates based on the size of nodal metastases, with micrometastatic disease showing better outcomes, supporting the prognostic value of this refinement^17^. Furthermore, the new differentiation of ovarian metastasis into Stage IA3 and Stage IIIA1 addresses a long-standing challenge in EC staging. Stage IA3 now classifies low-grade ECs with specific favorable criteria involving the uterus and ovary, recognized for their excellent prognosis, often leading to de-escalation of adjuvant therapy. Conversely, Stage IIIA1 encompasses other metastatic patterns to the ovary with a worse prognosis. Gravbrot et al. reported a significantly higher 10-year OS for 2023 Stage IA3 (73.4%) compared to 2009 Stage IIIA (52.6%)^14^. This distinction prevents the over-staging of favorable cases, allowing for a more accurate delineation of Stage III from earlier disease, and therefore enhancing the clarity between intermediate and advanced stages. Our findings further support this refinement, as the four patients reclassified from Stage IIIA–IA3 in our cohort exhibited excellent outcomes, with a 100% 5-year overall survival.

The integration of aggressive histological types and substantial LVSI into the staging system affects the classification of early-stage disease, leading to upstaging of high-risk Stage I cases to Stage II. Aggressive histologies with myometrial invasion are classified as IIC, while non-aggressive types with substantial LVSI are classified as IIB^1^. Schwameis et al.^18^ reported that 90% of early-stage shifts were upshifts, largely due to aggressive histological subtypes or p53 abnormalities, and that these IIC cases demonstrated substantially worse 5-year PFS and OS than their previous Stage IA/IB groupings. This recalibration effectively separates risk groups that were previously blurred under the 2009 system, leading to a more robust prognostic model overall. Our data confirm this pattern, with 28 patients upstaged from IA to IIC demonstrating 5-year OS of 71.4% compared with 93.8% for those remaining in Stage IA1, validating the prognostic relevance of this reclassification.

The improved prognostic discrimination demonstrated by the FIGO 2023 system has implications for treatment decision-making, although we cannot assess this directly in our retrospective cohort, where all treatment was based on 2009 staging. Current guidelines recommend distinct therapeutic approaches based on the refined 2023 classifications. Stage IIC patients, particularly those with aggressive histology and myometrial invasion, are increasingly considered for platinum-based chemotherapy in addition to radiation, representing treatment intensification compared with historical Stage IA management. Conversely, Stage IA3 patients with lowgrade disease and favorable ovarian involvement may forego adjuvant therapy entirely in selected cases, representing appropriate de-escalation. The disaggregated Stage IV subgroups also inform differential treatment intensity, with Stage IIIB2 patients potentially benefiting from aggressive locoregional therapy, including cytoreductive surgery and radiation, while Stage IVC patients with distant metastases are managed with systemic chemotherapy. The enhanced prognostic separation we demonstrate suggests these treatment modifications are appropriately matched to underlying risk.

While our study provides valuable insights, it is not without limitations. The retrospective nature of the study and the single-institution design may limit the generalizability of our findings. The absence of molecular profiling data for our cohort is a significant limitation given the emphasis on molecular classification in the 2023 FIGO system. Our use of the Mayo criteria for selective lymph node assessment, while reasonable during the 2008–2018 treatment period, is not the most contemporary standard. Sentinel lymph node mapping, which has become increasingly adopted, may provide more accurate nodal staging and could have altered stage classification in some patients. Owing to the retrospective nature of the study, we were unable to evaluate how stage reclassification under FIGO 2023 might have influenced treatment selection or surveillance strategies, and therefore cannot directly attribute observed survival differences to treatment-related factors. All therapeutic decisions in this cohort were based on the FIGO 2009 staging system in use at the time. Prospective studies in which treatment is explicitly guided by FIGO 2023 staging are warranted to more fully elucidate the clinical impact of this revised classification system. Nevertheless, our study contributes to the growing body of evidence supporting the clinical relevance and superior prognostic capabilities of the 2023 FIGO staging system, advocating for its widespread adoption in clinical practice to optimize patient management and improve outcomes in EC.

## CONCLUSION

Our findings confirm that the FIGO 2023 staging system provides improved prognostic discrimination compared with FIGO 2009, particularly in distinguishing Stage II from Stages III–IV disease. By integrating adverse histopathological features and molecular classifiers, the revised system offers a more precise framework for risk stratification and treatment planning. These results, together with growing international validation, strongly support the adoption of FIGO 2023 as the preferred staging system for EC.

## Data Availability

The datasets generated and/or analyzed during the current study are available from the corresponding author upon reasonable request.
