# Supplementary material for: Validation of the 2023 International Federation of Gynecology and Obstetrics staging system in a large retrospective cohort
Source: Rev Assoc Med Bras (1992). 2026 Jun 26;72(3):e20251624. doi: 10.1590/1806-9282.20251624 (PMC13316865; doi:10.1590/1806-9282.20251624)
Supplement: Supplementary Figure 1 [file 1806-9282-ramb-72-3-e20251624-suppl1.docx]

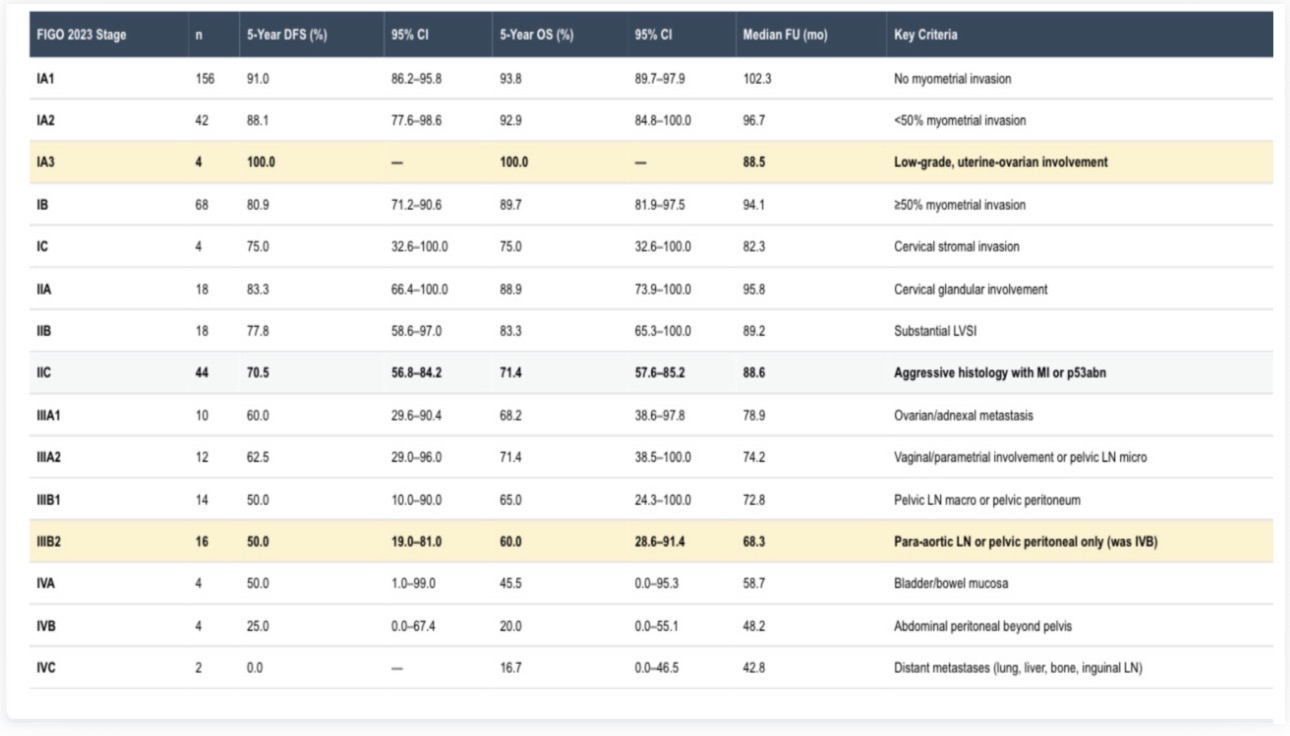


**Supplementary Figure 1.** Kaplan-Meier analysis of detailed survival outcomes for International Federation of Gynecology and Obstetrics 2023 sub-stage classification.
